# Active Pediatric Systemic Lupus Erythematosus in Fars Province, Iran: Associations With Geographical and Meteorological Determinants—A Retrospective Cross‑Sectional Study

**DOI:** 10.1002/hsr2.71755

**Published:** 2026-01-26

**Authors:** Zahra Kanannejad, Walter Robert Taylor, Koorosh Nikaein, Seyed Hesamedin Nabavizadeh, Soheila Alyasin, Ali Ghorbanpour, Mohammad Amin Ghatee

**Affiliations:** ^1^ Allergy Research Center Shiraz University of Medical Sciences Shiraz Iran; ^2^ Mahidol Oxford Tropical Medicine Research Unit Bangkok Thailand; ^3^ Centre for Tropical Medicine and Global Health, Nuffield Department of Medicine University of Oxford Oxford UK; ^4^ Student Research Committee Yasuj University of Medical Sciences Yasuj Iran; ^5^ Department of Allergy and Clinical Immunology Namazi Hospital Shiraz Iran; ^6^ School of Medicine Shiraz University of Medical Sciences Shiraz Iran; ^7^ Professor Alborzi Clinical Microbiology Research Center Shiraz University of Medical Sciences Shiraz Iran

**Keywords:** active pediatric systemic lupus erythematosus, climatic factors, geographical factors, GIS, rainfall, urban setting

## Abstract

**Background and Aims:**

Pediatric systemic lupus erythematosus (SLE) is a complex autoimmune disease influenced by genetic and environmental factors such as sunlight and temperature. This retrospective study investigated the association between meteorological and geographical factors and active pediatric SLE in Fars province, southwest Iran.

**Method:**

The residential addresses of pediatric patients with active SLE who were hospitalized at the main referral hospital of southwest Iran in Shiraz City between March 2016 and January 2023 were extracted from their medical records and geographically mapped. The influence of meteorological factors, such as temperature, humidity, evaporation, rainfall, as well as geographical parameters including land cover, slope, and altitude, on active pediatric SLE was evaluated using geographic information system (GIS) analysis. Data were analyzed using univariate and multivariate binary logistic regression analysis.

**Results:**

This study included 143 pediatric patients with active SLE from 34 out of a total of 8181 city/village areas. There was a significant positive association between urban setting and active pediatric SLE (OR = 75.949, CI = 9.521–605.846) while mean annual rainfall demonstrated a significant negative association (OR = 0.997, CI = 0.994–1.000) in the univariate analysis. Multivariate analysis revealed that urban setting was the only significant factor positively correlated with active pediatric SLE (OR = 56.567, CI = 6.731–745.372). There was no association between other meteorological and environmental factors and active pediatric SLE.

**Conclusion:**

This study found that living in urban areas and lower annual rainfall are significant risk factors for active pediatric SLE in Fars province, probably due to increased exposure to pollutants and ultraviolet radiation. While previous studies have linked SLE activity to temperature and humidity, no such associations were observed here, highlighting the need for further research on regional environmental influences on SLE.

## Summary

- Urban environment as a risk factor: Living in urban areas significantly increases the risk of active pediatric systemic lupus erythematosus (SLE).
- Protective role of rainfall: Higher mean annual rainfall is associated with a lower likelihood of active pediatric SLE, possibly due to its effect on reducing pollution and UV exposure.
- First GIS‐based study in Iran: This study uniquely applies Geographic Information System (GIS) analysis to investigate environmental influences on pediatric SLE in Fars province.

## 1 Introduction

Systemic lupus erythematosus (SLE) is a chronic autoimmune condition affecting multiple systems. The etiology of SLE remains unclear but it is believed to result from interactions between genetic and environmental factors [1]. Environmental factors play a crucial role in the development of SLE by inducing epigenetic changes, immune dysregulation, and loss of immune tolerance, ultimately leading to the onset or recurrence of the disease [2]. Ultraviolet radiation (UVR), lifestyle behaviors, occupational exposure to crystalline silica, exogenous estrogens, air pollution are the most significant environmental factors linked to SLE [2, 3]. In addition, climatic factors and geographical location may also contribute to the risk of developing SLE [4, 5].

Studies have shown that climate factors, such as temperature, atmospheric pressure, sunshine, humidity, wind speed, and precipitation, correlate with the activity and incidence of SLE [6, 7, 8]. Environmental factors can influence the progression of SLE through various mechanisms, including the regulation of inflammatory mediators, immune cell recruitment (dendritic cell (DC), macrophage, and neutrophil), and suppression of immune tolerance [9, 10]. For example, exposure to UVR increases the production of pro‐inflammatory cytokines, including interferon‐alpha (IFN‐α), interleukin (IL)‐1, IL‐6, and tumor necrosis factor‐alpha (TNF‐α) [11]. Low temperature has also been linked to the occurrence, development, and recurrence of SLE through the production of pro‐inflammatory cytokines [12, 13].

In addition to climatic factors, the effect of geographical variability on the distribution of SLE has also been assessed. Geographical factors, including rural versus urban settings, medical resources, and altitude, have been found to influence the prevalence, disease activity, and organ involvement in SLE patients [14, 15, 16]. Some studies indicate that SLE prevalence tends to be higher in urban areas compared to rural settings [17, 18, 19]. This difference may be attributed to increased environmental exposures (e.g., pollution, industrial toxins) and lifestyle factors such as reduced physical activity and dietary changes in urban areas. Moreover, in rural settings, a lack of medical infrastructure and trained professionals often results in delayed or inadequate treatment, contributing to worse disease outcomes. Some studies also proposed high altitude as a risk factor for active SLE, as hypoxic conditions can alter cytokine profiles, potentially influencing disease activity [16]. In addition, higher altitudes are associated with increased UV radiation, a known trigger for SLE flares [20].

Geographic information systems (GIS) are a valuable tool for investigating the influence of geographical and meteorological factors on disease development. GIS allows for the integration of diverse data sources to map specific diseases in relation to their environment and health infrastructure [21] There are limited data exploring the relation between SLE and geo‐climatic factors using GIS.

Williams and colleagues conducted a study using GIS to examine the effects of a dangerous waste site on the prevalence of lupus and autoimmune illnesses in the nearby community of Buffalo, New York [22]. They reported that individuals affected by SLE often experienced symptoms and were diagnosed with SLE during periods when the site was inactive, suggesting that chronic exposure to waste may have played a more significant role than acute triggers. Another study by Al‐Maini et al. examined the spatial distribution of incident SLE cases in Canada using GIS. Their analysis indicated that while ethnicity alone did not increase the risk of SLE, the combination of ethnicity and residential location significantly increased the risk of developing the disease [23].

## 2 Objectives

The aim of this study was to examine the association between active pediatric SLE and geographical parameters (land cover, altitude, slope) as well as meteorological factors (temperature, humidity, sunshine, rainfall, and evaporation), using GIS in Fars province, Iran, from March 2016 to January 2023. To the best of our knowledge, this is the first GIS‐based study in Iran investigating geographical and meteorological risk factor for active pediatric SLE.

## 3 Methods and Materials

### 3.1 Patients

This retrospective study was conducted on a cohort of 143 patients with active childhood SLE who were hospitalized at Nemazee Hospital in Shiraz, Iran, between March 2016 and January 2023. As the leading respiratory hospital in Fars province, Nemazee admits patients from diverse socioeconomic backgrounds across the province. Lupus disease activity was evaluated by the pediatric rheumatology team according to the European Alliance of Associations for Rheumatology (EULAR) recommendations [24]. Active SLE was defined as the presence of new, recurrent, or worsening clinical and/or laboratory manifestations attributable to lupus that indicated ongoing inflammatory disease requiring treatment modification. Consistent with EULAR guidance, classification of active disease incorporated: constitutional symptoms (e.g., fever, fatigue, weight loss); new or worsening mucocutaneous, musculoskeletal, renal, neuropsychiatric, hematologic, or serosal involvement; and laboratory abnormalities compatible with active SLE, including low complement levels, elevated anti–double‑stranded DNA antibodies, and/or increased inflammatory markers. No formal numerical disease activity index (such as SLEDAI‑2K) was applied; activity was assessed using EULAR‑based clinical judgment criteria routinely employed in pediatric rheumatology practice at our center.

The residential address of each patient was retrieved from their medical records. Patients with incomplete address information or those residing outside of Fars province were excluded from the study. The Ethics Committee of Shiraz University of Medical Sciences approved this study (Ethic no: IR.SUMS.REC.1401.579). All research was performed in accordance with relevant guidelines/regulations of Shiraz University of Medical Sciences. Patients' data were retrieved from Health Center records anonymously and under ethics committee rules. No clinical sample was taken from patients. Due to the retrospective nature of the study, the Ethics Committee of Shiraz University of Medical Sciences waived the need of obtaining informed consent.

### 3.2 Study Area

The study was conducted in Fars province, located in southwest Iran, within the geographical coordinates of longitude 27°31′ N and 31°42′ N and latitude 50°37′ E and 55°38′ E. The province has a population of approximately 4,851,274 people and covers an area of 122,608 km^2^. The region consists of 24 counties with a total of 8181 residential areas, including both small and large villages, with Shiraz as the capital city. Fars Province is characterized by diverse geographical features such as plains, deserts, lakes, mountains, valleys, and rivers. The climate in this province varies across three regions, with mountainous areas experiencing cold winters, the central area having moderate winters and hot summers, and other areas having mild winters and very hot summers.

### 3.3 Meteorological and Geographical Data

Data on meteorological parameters, including temperature, maximum and minimum temperature, humidity, maximum and minimum humidity, evaporation, sunny hours, and the number of rainy and frosty days, were collected from 27 synoptic meteorological stations across Fars Province. Additionally, rainfall data were collected from 86 rain‐gauge stations. All of these data were acquired from the Fars Province Weather Bureau, thus, providing a comprehensive picture of the region's climate and weather patterns. The mean values of all data were calculated every month for the duration of the study (March 2016 and January 2023). The annual iso‐hydral, iso‐humid, and frost days' raster layers were generated using the Kriging interpolation method, and iso‐thermal, iso‐evaporation, and rainy day' raster layers used the tension‐based Spline interpolation model with a resolution grid of 1 × 1 km.

Residential locations of all patients were obtained from their medical records and placed on a map using latitude and longitude data. Land cover vector layers and a Digital Elevation Model (DEM) raster layers were obtained from the Department of Natural Resources in Fars Province. A slope raster map was generated using the spatial analyst tool that used the DEM rater data to calculate the maximum rate of change in value between each cell and its neighboring cells. The land cover layer was used to provide spatial information on the diverse physical attributes of the province's surface.

### 3.4 Geospatial Analysis

Geographical and meteorological information were analyzed by Arc GIS version 10.5 [25]. The raster layer values were extracted to the point shape file layer for villages and cities. The identity tool was employed to calculate the geometrical intersection of the point layer derived from the extraction of all raster layers that, with the land cover (polygonal) vector layer, generated the final layer, with each point corresponding to the properties of all the merged identity features from the previously mentioned raster and vector layers. The final layer's property was changed to Excel format for statistical analysis.

### 3.5 Statistical Analysis

We used descriptive statistics to describe patient demographic features. The point layers of cities and villages of the studied counties were extracted with the raster layers, and then the geometric intersections of the obtained layer and land cover vector layer were computed by the identity tool to generate the final layer. Each point represented geographical and climatic values of all overlapped raster and vector layers in the final layer. We assessed the association between geographical and climatic factors and active SLE based on the spatial description of patients in Fars province. We extracted residential point data, including both active SLE‐reported and non‐reported villages and cities, from the final province villages/cities' point layers and analyzed them using univariate and multivariate logistic regression models. We performed the statistical analyses using SPSS version 21.

## 4 Results

This study identified 143 patients with active pediatric SLE from 34 out of a total of 8181 residential points. The patient population had a mean age of 13.85 ± 0.32 years, comprising 17.5% males (*n* = 25) and 82.5% females (*n* = 118). The mean duration of hospitalization was 5.54 ± 0.61 days, with a mean number of hospitalizations of 6.48 ± 0.48 over the study time frame of 93 months.

### 4.1 Active Pediatric SLE Distribution Based on Geographical and Meteorological Factors

**Figure 1:**
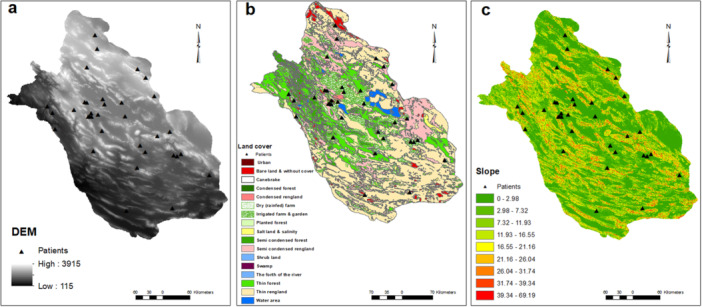
Patient distribution in Fars province with different elevation (a), land cover (b), and slope (c). Points with active pediatric SLE are shown by triangle symbols. Abbreviation: DEM, digital elevation model.

**Figure 2:**
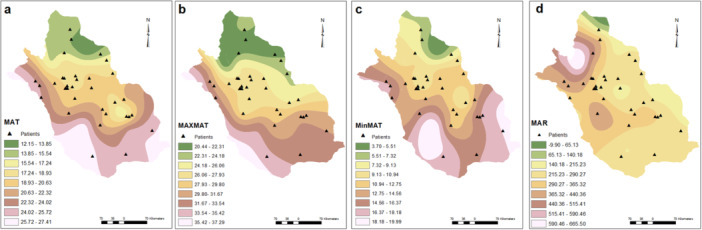
Patient distribution as functions of temperature and rainfall. MAT (a), maxMAT (b), minMAT (c), and MAR (d). MAR, mean annual rainfall; MAT, mean annual temperature.

**Figure 3:**
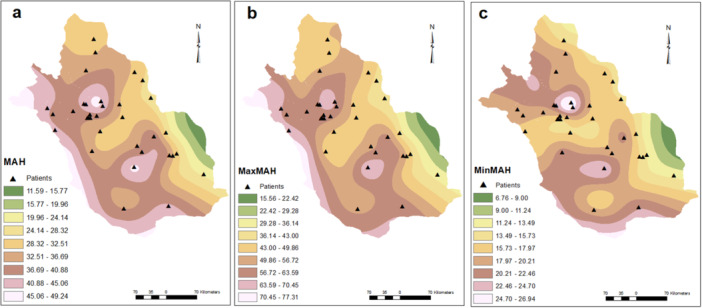
Patient distribution as functions of humidity. MAH (a), maxMAH (b), and minMAH (c). MAH, mean annual humidity.

**Figure 4:**
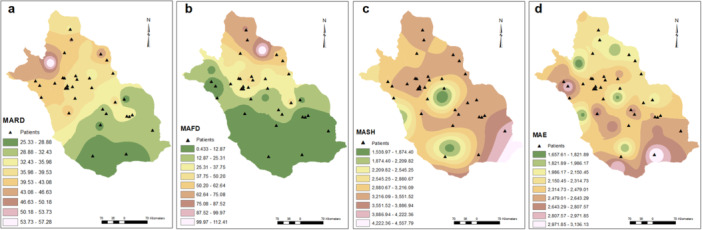
Patient distribution as functions of rainy, frosty days, sunny hours, and evaporation. MARD (a), MAFD (b), MASH (c), and MAE (d). MAFD, mean annual frosty day; MARD, mean annual rainy day; MASH, mean annual sunny hours; MAE, mean annual evaporation.

Active pediatric SLE patients lived in diverse residential areas across Fars province and the geographical and meteorological features are shown in Figures 1, 2, 3, 4.

### 4.2 Univariate Analysis

To clarify the comparison groups, all logistic regression models compared residential locations in which active pediatric SLE cases were identified (*n *= 34) versus all other residential locations with no active SLE cases (*n *= 8147). Thus, each OR reflects the change in odds of a location belonging to the active‐SLE group per unit change in the predictor, or relative to the reference category for categorical variables. The univariate analysis showed that an increasing mean annual rainfall was protective (*p *= 0.05) against active SLE: OR (odds ratio) = 0.997 (95% CI (confidence interval) = 0.994–1.000) as the only significant meteorological parameters associated negatively with active pediatric SLE (Table 1). Each unit increase in rainfall decreased the probability of active SLE by 0.3%. All other meteorological factors were not significant.

**Table 1:** The impact of meteorological and geographical parameters on active pediatric SLE.

Factors	*p* value	OR	CI
MAT	0.21	0.943	0.86–1.0340
minMAT	0.26	0.948	0.865–1.039
maxMAT	0.45	0.968	0.891–1.052
MAR	0.05	0.997	0.994–1.000
MAE	0.64	1	0.999–1.002
MAH	0.93	1.002	0.948–1.060
maxMAH	0.78	1.005	0.970–1.041
minMAH	0.61	0.974	0.881–1.077
Frosty days	0.48	1.005	0.992–1.018
Rainy days	0.96	1.001	0.947–1.059
Sunshine	0.14	1.001	1.000–1.002
Land cover			
Thin forest (constant)	*p* < 0.001		
Semi‐condensed forest	*p* > 0.99	0.000	0.000
Condensed forest	*p* > 0.99	0.000	0.000
Thin rangeland	0.45	2.250	0.271–18.720
Semi‐condensed rangeland	0.93	1.131	0.071–18.119
Condensed rangeland	*p* > 0.99	0.000	0.000
Dry farm	0.06	9.904	0.893–109.856
Irrigated farm and garden	0.21	3.664	0.483–27.775
Urban	*p* < 0.001	75.949	9.521–605.846
Bare and sandy land	*p* > 0.99	0.000	0.000
Water area and river forth	*p* > 0.99	0.000	0.000
DEM	0.59	1.000	1–0.001
Slope	0.73	1.000	0.999–1.002

Abbreviations: DEM, digital elevation model; MAE, mean annual evaporation; MAH, mean annual humidity; MAR, mean annual rainfall; MAT, mean annual temperature; min, minimum; max, maximum.

Among the geographical factors (Table 1), only living in an urban setting was significantly (*p* < 0.001) associated with an increased odd of active pediatric SLE: OR = 75.949 (95% CI: 9.521–605.846).

### 4.3 Multivariate Analysis

In the multivariate analysis, only urban setting (*p *< 0.001, OR = 56.567, CI = 6.731–745.372) was independently associated with an increased odd of SLE activity (Table 2).

**Table 2:** Multivariate analysis of geographical and meteorological factors associated with active pediatric SLE.

Factors	*p* value	OR	CI
MAR	0.26	0.998	0.994–1.002
Thin forest (constant)	*p* < 0.001		
Semi‐condensed forest	*p* > 0.99	0.000	0.000
Condensed forest	*p* > 0.99	0.000	0.000
Thin rangeland	0.67	1.617	0.182–14.381
Semi‐condensed rangeland	0.95	0.920	0.056–15.052
Condensed rangeland	*p* > 0.99	0.000	0.000
Dry farm	0.08	8.829	0.791–98.561
Irrigated farm and garden	0.33	2.790	0.351–22.177
Urban	*p* < 0.001	56.567	6.731–745.372
Bare and sandy land	*p* > 0.99	0.000	0.000
Water area and river forth	*p* > 0.99	0.000	0.000

Abbreviation: MAR, mean annual rainfall.

## 5 Discussion

Genetic factors are significant contributors to the development of SLE but environmental/meteorological factors also play a critical role in the occurrence of SLE and its development. Several studies have demonstrated an association between activity and incidence of SLE and temperature, humidity, UVR, and sunshine [6, 13, 26, 27]. Geographical parameters like altitude, longitude, and latitude are also risk factors for SLE development [16, 28]. In our study, the first in Fars province, we study found that active pediatric SLE was negatively linked to rainfall, while it was positively associated with living in an urban setting.

The association between rainfall and active SLE has been rarely examined. Lower precipitation is often linked to higher exposure to UVR from sunlight and UVR is a known trigger for SLE flares because it can induce inflammation, recruit immune cells (DC, macrophage, and neutrophil cells), suppress immune system tolerance, and promote B‐ and T‐cell activation, giving rise to the development of SLE and exacerbating current disease [9]. Furthermore, rainfall has the potential to reduce air pollutants, notably, particular matter (PM) 2.5, nitrogen dioxide (NO_2_), and sulfur dioxide (SO_2_), which are known to increase the risk of SLE relapse and hospital admissions [29, 30]. Particular matter includes sulfates, nitrates, black carbon, and silica, which enter the bloodstream through the alveoli. Studies have demonstrated that particulate matter can activate the body's natural defense mechanisms by influencing the signaling of Toll‐like receptors (TLRs) and the activation of inflammasomes [31]. TLRs and inflammasome activation are crucial factors in the development of SLE and other autoimmune disorders [32]. A recent study conducted on individuals with SLE in the Baltimore (USA) observed an increase in occurrences of joint and rash flares correlated with elevated levels of ambient PM2.5 [33]. whilst another group found a correlation between PM2.5, levels of anti‐DS DNA antibodies, and the presence of urine cellular casts in individuals with active SLE [34]. It is likely that an increase in PM2.5 caused by catastrophic wildfires may adversely affect individuals with SLE, suggesting that rainfall may indirectly benefit SLE patients by reducing exposure to certain environmental triggers.

In contrast with our findings, some studies have found a positive association between rainfall and active SLE. Yang et al. showed positive correlation between the amount of precipitation and the number of active SLE patients in Anhui, China [6]. They hypothesized that individuals with SLE may be more susceptible to disease flare‐ups when living in humid environments that decreased immune function and lead to increased vulnerability to bacterial infections. However, one study reported no association between rainfall and SLE [13].

The current study detected the urban setting as a geographical risk factor for active SLE in childhood, consistent with several studies. A cross‐sectional analysis performed to study SLE risk, manifestations, and severity in relation to urban versus rural residence in Crete, Greece [17], found an increased prevalence in urban areas [17] whilst a Canadian study found that living in urban areas was associated with an increased risk of autoimmune rheumatic diseases, including SLE [18]. A case‐control study in urban neighborhoods of Boston with large African‐American populations found an increased risk of SLE in the urban population [19], consistent with a study from northwest Greece by Alamanos et al. [35] There are several potential reasons for the higher incidence of SLE in urban areas compared to rural areas. Urban areas are often associated with higher levels of environmental pollutants, such as air pollution from motor vehicles, which have been linked to an increased risk of autoimmune diseases like SLE [36]. Genetic predisposition, combined with lifestyle factors prevalent in urban environments, may contribute to the higher prevalence of SLE [36]. Socioeconomic factors associated with urban living, such as access to healthcare, stress levels, and lifestyle choices, can also influence the prevalence of SLE [37]. Disparities in healthcare access and quality, as well as higher stress levels commonly found in urban populations, may contribute to the higher prevalence of autoimmune diseases like SLE [38]. Urban areas may have higher levels of exposure to UVR, which have been linked to the development of SLE [36].

By contrast, several studies have identified rural residency as a potential risk factor for SLE. In a multiethnic Latin American cohort, Pons‐Estel et al. reported that rural residency was associated with higher level of SLE activity at diagnosis with renal involvement [39]. A case‐control study in Egypt found that living near agricultural areas was associated with ~12‐fold higher risk of developing SLE compared to those not living in these areas [40]. These studies hypothesized that poorer outcomes in rural SLE patients, lower socioeconomic status and education levels, poorer access to specialized rheumatology care may be the reasons for the poorer outcomes in rural SLE patients.

Temperature, humidity, and sunshine duration were identified as common meteorological risk factors for active SLE in some studies [13, 27]. However, we cannot find significant associations between these factors and active SLE. Temperature variations, particularly lower temperatures, may increase the activity of SLE [6, 13, 27, 41] via an increase in pro‐inflammatory cytokines, such as TNF‐α, IL‐6, and IL‐12, which are produced by monocytes [42, 43]. Furthermore, exposure to cold triggers the production of the inflammatory adhesion molecules E‐selectin, intercellular adhesion molecule‐1 (ICAM‐1), and vascular cell adhesion molecule‐1 (VCAM‐1), and activates complement [44, 45]. Sunshine exposure and UVR also induce a pro‐inflammatory environment that triggers abnormal and persistent photo reactivity through the activation of pro‐inflammatory cytokines, chemokines, and adhesion molecules [9, 10, 46]. Several epidemiological factors have found a positive association between sunshine exposure and active SLE [27, 47, 48, 49]. High humidity has also been reported as another potential risk factor for active SLE in some epidemiological studies [28, 50] and this may be related to reduced immune function or modulating gut microbiota [51].

An important limitation of this study is the relatively small number of pediatric SLE cases (*n* = 143) in relation to the large number of geographical and meteorological predictors evaluated. Also, it should be noted that our study tried to included almost all active pediatric SLE in the Fars Province. Another important limitation of this study is the absence of validated, spatially resolved air pollution measurements (e.g., PM₂.₅, NO₂, SO₂) corresponding to the study period and residential locations of patients. Although rainfall was inversely associated with active pediatric SLE and is hypothesized to exert a protective effect partly through the reduction of ambient air pollutants, our data set did not include validated spatially resolved pollution measurements for the study period (2016–2023). High‑quality pollution data at the village/city level across Fars province were not available from the Department of Environment or the national meteorological database, and historical monthly pollution layers could not be reliably interpolated for the full province due to insufficient monitoring stations.

## 6 Conclusions

This retrospective study detected low rainfall and urban setting as potential risk factors associated with active pediatric SLE in Fars province, Iran. Further research is needed to elucidate the mechanisms by which these environmental exposures impact disease presentation and severity in pediatric lupus.

## Author Contributions

M.A.G. and Z.K. were involved in conception and design. K.N. and A.G. were responsible for acquisition of data. M.A.G. performed analysis and interpretation of data. Z.K. was involved in drafting the article. W.R.T., S.A., and H.N. were involved in revising the article. All authors approved the final version to be published. The corresponding author (M.A.G.) had full access to all of the data in this study and takes complete responsibility for the integrity of the data and the accuracy of the data analysis.

## Ethics Statement

This study was approved by Shiraz University of Medical Sciences (Ethic No: IR.SUMS.REC.1401.579). Current project is a retrospective study and patients' data were retrieved from Health Center sheet anonymously and under ethics committee rules. No clinical sample was taken from patients.

## Conflicts of Interest

The authors declare no conflicts of interest.

## Transparency Statement

The corresponding author, Mohammad Amin Ghatee, affirms that this manuscript is an honest, accurate, and transparent account of the study being reported; that no important aspects of the study have been omitted; and that any discrepancies from the study as planned (and, if relevant, registered) have been explained.

## Data Availability

The data that support the findings of this study are available on request from the corresponding author. The data are not publicly available due to privacy or ethical restrictions.
